# Bio-inspired facile fabrication of silver nanoparticles from *in vitro* grown shoots of *Tamarix nilotica*: explication of its potential in impeding growth and biofilms of *Listeria monocytogenes* and assessment of wound healing ability

**DOI:** 10.1039/d0ra04587j

**Published:** 2020-08-17

**Authors:** Nasser A. Al-Shabib, Fohad Mabood Husain, Mohammad Nadeem, Mohd Shahnawaz Khan, Fahad Al-Qurainy, Abdullah A. Alyousef, Mohammed Arshad, Altaf Khan, Javed Masood Khan, Pravej Alam, Thamer Albalawi, Syed Ali Shahzad

**Affiliations:** Department of Food Science and Nutrition, Faculty of Food and Agricultural Sciences, King Saud University 2456 Riyadh 11451 Kingdom of Saudi Arabia nalshabib@ksu.edu.sa fhusain@ksu.edu.sa; Department of Botany and Microbiology, College of Science, King Saud University 2456 Riyadh 11451 Kingdom of Saudi Arabia; Department of Biochemistry, College of Science, King Saud University 2456 Riyadh 11451 Kingdom of Saudi Arabia; Department of Clinical Laboratory Sciences, College of Applied Medical Sciences, King Saud University 2460 Riyadh 11451 Kingdom of Saudi Arabia; Department of Pharmacology and Toxicology, Central Laboratory, College of Pharmacy, King Saud University 2460 Riyadh 11451 Kingdom of Saudi Arabia; Department of Biology, Prince Sattam bin Abdulaziz Univrsity Alkharj Kingdom of Saudi Arabia

## Abstract

Novel, safe, and effective antilisterial agents are required in order to prevent *Listeria monocytogenes* infections and maintain food safety. This study synthesized silver nanoparticles (AgNPs) from the shoot extract of *in vitro*-grown *Tamarix nilotica* (TN) and characterized them using X-ray diffraction, Fourier transform infrared spectroscopy, UV-visible spectroscopy, dynamic light scattering, scanning electron microscopy (SEM), energy-dispersive X-ray spectroscopy (EDXS), and transmission electron microscopy (TEM). We also assessed the antilisterial potential of the synthesized TN-AgNPs by determining the minimum inhibitory concentration (MIC) and minimum bactericidal concentration (MBC) against two strains of *L. monocytogenes* and *L. innocua*. TN-AgNPs (2×MICs) showed a significant decrease in growth in all *Listeria* test strains. Release of cellular content and cell morphology analysis of TN-AgNP-treated bacterial cells demonstrated the mechanism of bactericidal activity of AgNPs. In addition, TN-AgNPs induced a significant decrease in swimming motility (62–71%), biofilm formation (57–64%), and preformed biofilms (48–58%) in all *Listeria* test strains at sub-inhibitory concentrations. Microtitre plate assay results for biofilm inhibition were confirmed by SEM and CLSM visualization of TN-AgNP-treated and TN-AgNP-untreated *Listeria* test strains. TN-AgNPs also showed wound-healing activity in MCF-7 cells by inhibiting cell migration in a scratch plate assay. TN-AgNP-induced enhanced reactive oxygen species generation in treated cells could be a plausible reason for the biofilm inhibitory activity of AgNPs. TN-AgNPs having antilisterial, antibiofilm, and wound-healing properties can be effectively used to prevent *L. monocytogenes* infections in the food industry and healthcare.

## Abstract 2

Novel, safe, and effective antilisterial agents are required in order to prevent *Listeria monocytogenes* infections and maintain food safety.

## Graphical abstract

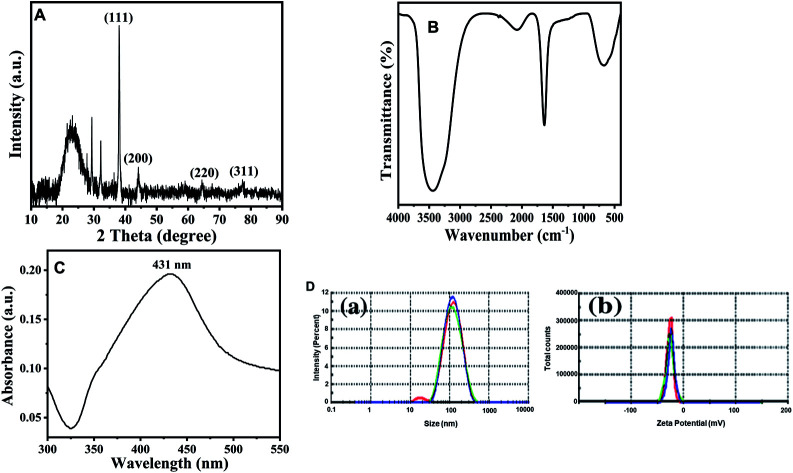

## 1. Introduction

*Listeria monocytogenes* is a key foodborne human pathogen and, because of its high pathogenicity, is considered one of the most harmful bacteria associated with the food industry. The most common sources of listerial infections are processed and ready-to-eat frozen foods.^1^ Listeriosis has a low incidence rate but can be lethal to susceptible populations, such as pregnant women, newborns, the elderly, and immunocompromised people; in immunocompetent people, listeriosis is non-invasive. It can cause mild gastroenteritis to potentially fatal infections, such as septicemia, encephalitis, and meningitis, depending on the individual's vulnerability.^2^

*Listeria* is ubiquitous in nature and so contaminates food during various stages of processing and distribution. Ready-to-eat foods are at high risk of *Listeria* infections because of a lack of effective decontamination methods that can destroy viable *L. monocytogenes* cells or because of recontamination post decontamination.^3,4^ In addition, *Listeria* spp. flourish at 0–4 °C, and their tolerance to commonly used food preservatives is a big challenge in maintaining food safety. Therefore, temperature control is of great importance in controlling *L. monocytogenes* growth in fast food, and even a small inoculum during production, transport, or retail or at the consumer level can lead to a significant pathogenic burden.^5^ Biofilm formation by *L. monocytogenes* on abiotic and biotic surfaces in a food-processing environment accounts for most of the contamination of processed foods.^6^ Decontamination of surfaces on which biofilm formation occurs is a tough challenge because bacteria in biofilms are more resistant to detergents, chemicals, and disinfectants compared to their planktonic forms. Biofilms help bacteria survive in unsuitable environmental settings such as acidity, low temperature, and high salinity.^7^ Therefore, biofilm formation helps *L. monocytogenes* withstand stress and enhances its viability in unfavorable conditions.^8^

The development of a novel, safe, and effective antilisterial agent is required in order to prevent *L. monocytogenes* infections and maintain food safety. Nanoparticles (NPs) possess unique physicochemical, catalytic, electronic, and biological properties and are used in biosensing, catalysis, drug delivery, cosmetics, and various biomedical applications.^9^ NP synthesis using chemical and physical methods is hazardous because huge amounts of chemicals are generated as by-products. In addition, NP synthesis is expensive, time consuming, and energy consuming.^10^ Green or bioinspired NP synthesis uses biological sources for producing metallic NPs through a rapid, economical, and eco-friendly process. Plant extracts and microbes are extensively used to produce NPs of desired shapes and sizes.^11,12^ However, culture time and cross-contamination of microbial cultures limit their use as substrates for NP synthesis. In contrast, the abundant availability of plant material makes it a most suitable candidate for NP synthesis.^13^ Almost all plant parts, ranging from roots, to leaves, peel, fruits, and seeds, can be used as reducing and capping agents for metallic NP synthesis.^14^ Plant substrates act as effective reductants because of the presence of an array of phytochemicals, proteins, antioxidants, and amino acids.^15^

Silver nanoparticles (AgNPs) have broad-spectrum bactericidal and fungicidal potential and do not induce resistance in bacteria.^16^ The antimicrobial activity of AgNPs is due to their high surface-area-to-volume ratio and slow, sustained release of Ag^+^ into microbial cells.^17^ However, there are few reports on the antibacterial and antibiofilm activity of AgNPs against *Listeria* spp.

*Tamarix nilotica* (Ehrenb.; TN) Bunge belongs to the Tamaricaceae family and is a native shrub of the Middle East and North Africa. In ancient Egypt, Bunge was used to treat fever, migraine, and irritation and as an antiseptic and sexual enhancer. Various parts of TN are used in traditional medicine; for example, cooked leaves and young branches are used for edema of the spleen, leaves blended in ginger are used for uterine diseases, and bark bubbled in water with vinegar is used as a moisturizer against lice.^18^ The plant's antioxidant activity due to the presence of various phenolic compounds is also well established.^19^

However, TN has not yet been studied for NP synthesis, and using such a medicinally important plant for NP synthesis would be an added advantage. Therefore, this study synthesized AgNPs from *in vitro*-grown shoots of TN and characterized them using X-ray diffraction (XRD), Fourier transform infrared (FTIR) spectroscopy, UV-visible spectroscopy, dynamic light scattering (DLS), scanning electron microscopy (SEM), energy-dispersive X-ray spectroscopy (EDX), transmission electron microscopy (TEM), and confocal laser scanning microscopy (CLSM) to assess their antilisterial and biofilm inhibitory potential against two strains of *L. monocytogenes* and one strain of *L. innocua*. We also investigated the plausible mechanism of action of AgNPs against *Listeria* spp. In addition, we explored the wound-healing potential of AgNPs using scratch plate assay.

## 2. Material and methods

### 2.1. *Listeria* strains

*Listeria monocytogenes* ATCC 19114, *L. monocytogenes* ATCC 13932 and *L. innocua* 33090 were used in present study. All cultures were stored at −80 °C and were grown in Tryptose Soy broth (TSB) (Oxoid, UK) at 37 °C.

### 2.2. Preparation of *Tamarix nilotica* shoots

Explants of *Tamarix nilotica* were washed for 10 min under running water, sterilized in ethanol 70% (v/v) for 1 min and plunged twice (10 min each) in Chlorox (3.0–4.0% accessible chloride) with 5–6 drops of Teepol. Thereafter, explant material was exposed to fungicide carbendazim (1%, Bayer, Turkey) for 10 min followed by three washes in sterile water (5 min each). Sterilized explants (1 cm long cuttings with axillary bud) were put on MS medium containing TDZ (thidiazuron) in thirty phyta jars with 50 ml medium containing five explants each. Cultures were incubated in a growth chamber at 25 ± 1 °C with a 16 h photoperiod and irradiance of 98 μmol m^−2^ s^−1^ enhanced by Phillips L36W/21 lights.^19^

### 2.3. Preparation of extract

*In vitro* grown shoots of *T. nilotica* were collected, washed thoroughly to remove all attached growth media and disinfected with ethanol (70%). Approx. 20 g of the shoots were dissolved in 100 ml of double distilled water and boiled for 5 min. The obtained extract was passed through Whatman No. 1 filter paper (Maidstone, UK) and centrifuged at 5000 rpm for 5 min to sediment out residual particles. This extract of *Tamarix nilotica* was used for the bio-fabrication of AgNPs.^20^

### 2.4. Bio-inspired synthesis of TN-AgNPs

Synthesis was initiated at room temperature by mixing the extract (1 ml) with 1 mM aqueous silver nitrate (20 ml) solution. The transformation of colorless suspension to dark brown color was indicative of the formation of NPs by reduction of AgNO_3_ to silver ion (Ag^+^). Centrifugation (10 000 rpm, 25 min) was done to harvest the AgNPs. Pellet was washed and dried overnight at 60 °C.^12^

### 2.5. Characterization of TN-AgNPs

The bio-fabricated AgNPs were characterized using X-ray diffraction (XRD) in the 2 theta range (10–80°) using a PANalytical diffractometer. Fourier transform infrared (FTIR) spectra was obtained in the range of 4000–400 cm^−1^ to assess the functional groups involved in the synthesis of nanoparticles. Water diluted samples of fabricated nanoparticles were scanned between 300-700 nm and absorption spectrum was recorded on a UV-visible spectrophotometer (ELICO SL-159). The average size of the particle, polydispersity index (PDI), and zeta potential of the nanoparticles were determined by dynamic light scattering (DLS) on a Zetasizer HPPS-5001 (Malvern, UK) at 25 °C at a scattering angle of 90°.

Further, morphological characterization of the fabricated AgNPs was accomplished using scanning electron microscopy (SEM) and transmission electron microscopy (TEM). SEM of silver nanoparticles was performed using, SEM, JSM-7001F, JEOL, Japan equipped with energy dispersive X-ray spectroscopy (EDS). Finely powdered silver nanoparticles were place onto sample holder and visualized under scanning electron microscope at different magnifications. Transmission electron microscopy (TEM) was performed using JEOL 2010, Japan. The aqueous suspension of silver nanoparticles was made in double distilled water and sonicated to make an even dispersion. Ten microliter of sample was placed on TEM grid and left to air dry overnight at room temperature. The images were captured at varying magnifications.

### 2.6. Antilisterial activity of TN-AgNPs

Antilisterial activity in terms of minimum inhibitory concentration (MIC) and minimum bactericidal concentration (MBC) of the AgNPs was determined using the microbroth dilution method.^21^ Briefly, 100 μl of *Listeria* cultures were added to each well of 96 well microtitre plate and two fold dilutions of AgNPs (concentrations ranging from 0.5–128 μg ml^−1^) were added to each well. Plates were incubated at 37 °C and observed for growth.

Growth kinetics studies at 2×MIC of TN-AgNPs were performed to further study the antibacterial activity against all the three strains of *Listeria* spp. Effect on growth was observed in terms of the optical density at 600 nm. Bacteria both untreated and treated (2×MIC) were grown in Tryptic soy broth (TSB) in 100 ml flasks and 1 ml were withdrawn at an interval of 2 h and optical density was measured.^12^

### 2.7. Loss of cellular content

Cellular content in the supernatant of TN-AgNPs treated and untreated cultures of test pathogen bacteria was determined by method described earlier.^22,23^ Overnight grown cultures of *Listeria* were centrifuged, washed thrice and suspended in MES buffer (pH 5.5). TN-AgNPs (2×MIC) were added to the suspension and incubated for 5 h, while suspension without the amendment of AgNPs were used as control. Suspension was passed through 0.22 μm filter and absorption was read at 260 nm.

### 2.8. Scanning electron microscopic (SEM) examination of cell surface morphology

Cell surface morphology of TN-AgNPs treated and untreated bacteria was observed under Scanning electron microscope (JSM-7001F, JEOL, Japan). Bacteria (1 ml) were centrifuged (5000 rpm, 10 min), resulting pellet was washed thrice. Subsequently, the washed cells were fixed with 2.5% glutaraldehyde and incubated overnight at 4 °C. Furthermore, samples were again washed three times and were post fixed with osmium tetraoxide for 60 min. After post fixation, cells were again rinsed and subjected to dehydration using graded ethanol series (25%, 50%, 70%, 90% and 100%). Dehydrated samples were gold coated by sputter coater and analyzed under SEM.

### 2.9. Swimming assay

Swimming motility of the test bacteria was assessed on Luria–Bertani (LB) containing 0.3% agar. AgNPs were amended to the agar medium to obtain desired sub-inhibitory concentrations against *L. monocytogenes* 19114, *L. monocytogenes* 13932 and. *L. innocua* 33090. NPs amended plates were dried at room temperature for half an hour and then 5 μl of the bacteria were point inoculated on the surface of their respective plates. Inoculated plates were incubated at 37 °C for 24 h. Diameter of the swim in the presence and absence of AgNPs was measured.^24^

### 2.10. Biofilm inhibition assay

#### 2.10.1. Effect on biofilm formation

Test pathogens *L. monocytogenes* and *L. innocua* were cultured overnight in TSB. Overnight grown bacteria were diluted in wells containing fresh Tryptic soy broth corresponding to approx. 1 × 10^9^ CFU ml^−1^, 0.5×MIC of TN-AgNPs was added to wells and incubated at 37 °C for a day. Post incubation, all wells were emptied to remove unattached cells and all wells were washed with sterile water. Subsequently, 1% crystal violet was used to stain bound cells and incubated for 15 min. Crystal violet was decanted and the wells were washed to remove excess stain. Crystal violet was dissolved in ethanol (200 μl) and absorbance was read at 585 nm to determine the biofilm inhibition. The percent inhibition of biofilms was calculated using below equation:
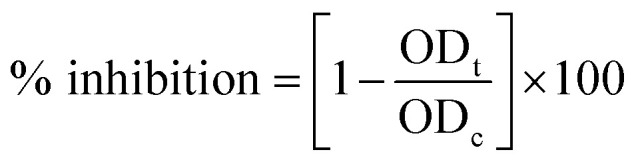
where, OD_t_ and OD_c_ are the absorbances of treated and control samples.

#### 2.10.2. Microscopic examination of biofilm structure

Biofilm architecture of TN-AgNPs treated and untreated test *Listeria* strains grown on glass coverslips at 37 °C for 24 h were visualized using SEM and confocal laser scanning microscopy (CLSM).^25^ For SEM, biofilm was developed on coverslips as mentioned above. Coverslips were stained with glutaraldehyde (2.5%) and subsequently dehydrated with a gradient of ethanol. Coverslips were gold coated by sputter coater and analyzed under SEM. Similarly, CLSM analysis was performed by staining the coverslip with 0.1% acridine orange in dark and visualizing under JEOL-JSM 6510 LV confocal laser scanning microscope.

#### 2.10.3. Disruption of mature (pre-formed) biofilms

Effect of 0.5×MIC of TN-AgNPs on pre-formed biofilms of *L. monocytogenes* 19114, *L. monocytogenes* 13932 and *L. innocua* 33090 was also studied. Test pathogens were allowed to grow in the wells of 96 well microtitre plate (MTP) for 24 h. Post incubation, all medium was aspirated out to remove unbound cells and fresh medium was added. TN-AgNPs (0.5×MIC) were added to wells except for the control and MTP was incubated for 24 h. Growth medium was decanted from the wells, cells were washed three times with sterilized distilled water and stained with 0.1% crystal violet. After removing excess stain ethanol (70%) was added and absorbance was recorded at 585 nm.^26^

### 2.11. ROS generation

Strains of *Listeria* growing exponentially were harvested and centrifuged to obtain metabolically active cells. Washed cells were added to fresh TSB medium, 2,7-dichlorofluorescein diacetate (DCFH-DA) was added and cells were left for incubation at shaking for half hour. Thereafter, cells were centrifuged and washed two times to remove the probe. These cells were dispensed into 96-well MTP and AgNPs (0.5×MIC) were amended in respective wells. Fluorescent intensity of AgNPs treated and control groups was recorded using fluorescence spectrophotometer (JASCO FP750).^27^

### 2.12. Wound healing assay: cell migration analysis

*In vitro* scratch/wound healing assay by culturing MCF-7 cells was performed to explore the effect of AgNPs on the cell–cell interaction, and cell migration aspects.^28^ Briefly, equal number (1 ×10^6^/well) of MCF-7 cells were seeded into respective wells of 24 well culture plates and grown to monolayer confluency. Thereafter, cells were treated with different concentration of AgNPs (0.5–10 μg ml^−1^). A scratch was made in the monolayer with the help of sterile pipette tip. Then unattached cells were decanted by washing with phosphate buffer saline (PBS). Images of control cells (culture medium) and treated cells after 0 h, 24 h and 48 h were captured on Leica microscope at 20× magnification.

### 2.13. Statistical analysis

All experiments were done in triplicates and mean values are presented as data. Level of significance was analyzed using Student's *t*-test.

## 3. Results

### 3.1. X-ray diffraction analysis of silver nanoparticles

**Fig. 1:**
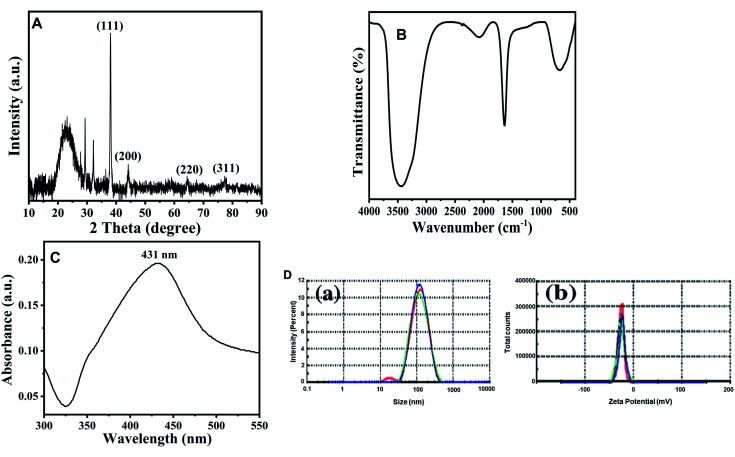
Characterization of TN-AgNPs. (A) XRD pattern of synthesized AgNPs. (B) FT-IR spectrum of synthesised AgNPs. (C) UV-visible absorbance spectra of as prepared AgNPs. (D) (a) particle size distribution; (b) zeta potential measurement of prepared AgNPs nanoparticles.

The crystalline nature of Ag NPs was confirmed by the examining of XRD pattern as shown in Fig. 1A. The XRD spectrum shows prominent peaks at 2*θ* = 38.14°, 44.12°, 64.70° and 77.43° representing (111), (200), (220) and (311) Bragg's reflections, respectively. It represents the *Fm*3*m* space group and face centered cubic system with lattice constant *α* = 4.08 Å. The diffraction peaks are broad which signifies the small crystalline size.^29^ The extra peaks near 23.18°, 29.37°, and 32.14° are due to the presence of organic phase on the silver particles. Peaks were matched with a database of Joint Committee on Powder Diffraction Standards (JCPDS) file no. 04-0783. The average size of the Ag nanoparticles was estimated by using the Debye–Scherrer equation^30^ and found to be around 81.32 nm.

### 3.2. FTIR analysis of silver nanoparticles

Identification of functional groups and its interaction with the Ag NPs was analyzed using FTIR spectrometer in the range of 400–4000 cm^−1^. The spectrum demonstrated that major absorption peaks were recorded at 3448, 2075, 1632, and 667 cm^−1^, signifying that the molecules of *T. nilotica* extraction act as capping agents that were bound on AgNPs. The absorption peak at region 3448 cm^−1^ was the reason for –OH stretching vibration of H bonded alcohol, water, phenols.^27^ The small peak band at 2075 cm^−1^ and 1631 cm^−1^ in the spectra corresponds to the C–N and C–C stretching vibration that contain functional group of nitriles and amines.^31^ The absorption band at 667 cm^−1^ is due to the presence of C–H bend alkynes as shown in Fig. 1B.

### 3.3. UV-visible spectroscopy analysis of silver nanoparticles

Fig. 1C shows the UV-visible spectrum of the dark-brown aqueous AgNP solution. Metal NPs have free electrons, which give a surface plasmon resonance absorption band in the visible range at 431 nm, indicating the formation of AgNPs.^32^

### 3.4. Dynamic light scattering (DLS) analysis of silver nanoparticles

DLS analysis was used to determine the hydrodynamic size, polydispersity index (PDI) and surface zeta potential of the synthesized AgNPs in aqueous environment. Green AgNPs had an average particle size of 131 nm, but the obtained size was higher than the TEM results (Fig. 1D(a)). The PDI value of AgNPs was 0.222, confirming the polydispersity of AgNPs; PDI < 0.1 typically indicates monodispersity. The surface zeta potential was negative (−23.6 mV), as shown in Fig. 1D(b). A high absolute value of the surface zeta potential specifies a high electrical charge on the NP surface, which can cause a strong repellent force among NPs to prevent agglomeration.^33^

### 3.5. Morphological analysis of synthesized AgNPs

Numerous biological agents such as plants and microbes (both bacteria and fungi) have been exploited by researchers for the bio-inspired fabrication of AgNPs.^34^ In the current investigation, the aqueous shoot extract of *Tamarix nilotica* was used to produce AgNPs. The addition of plant extract to AgNO_3_ solution resulted in gradual transformation of colorless solution to brown to dark black solution, indicating the formation of AgNPs.^35^

**Fig. 2:**
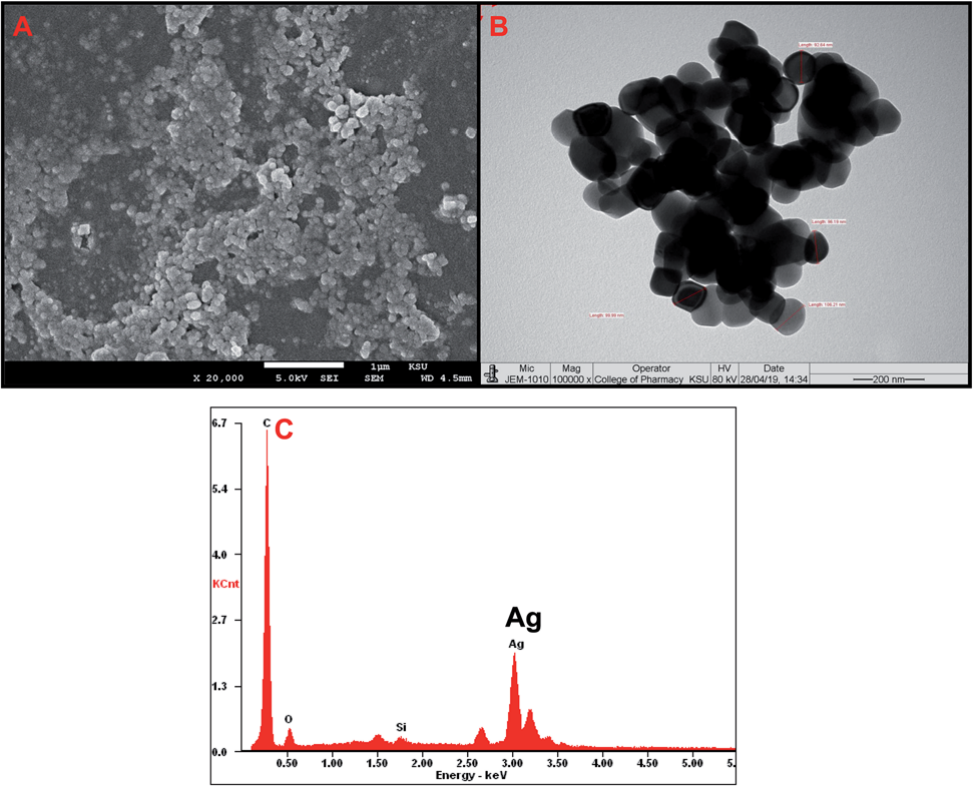
Characterization of AgNPs using electron microscopy. (A) Scanning electron microscopic (SEM) images of fabricated AgNPs visualized at 20 000×. (B) Transmission electron microscopic (TEM) images of fabricated AgNPs visualized at 100 000×. (C) EDX spectra of AgNPs.

Fig. 2A shows SEM micrograph of AgNPs. As evident from the figure, most of the particles appeared as spherical in nature, a few more spheroidal, and other varying shapes were also observed. The image clearly depicts that there a narrow size distribution with most of the particles of approximately same size. The size and range of silver nanoparticles was further validated using TEM and image is presented in Fig. 2B. The images clearly confirmed that most of particles were spherical with slight variation in size range. The size distribution using TEM was found to be in the 93–121 nm range.

The elemental compositions of AgNPs has been analyzed by EDS, and it is shown in Fig. 2C. The EDS spectrum showed a major peak at 3.0 keV, corresponding to Ag and confirming the presence of AgNPs. The EDS results also demonstrated the presence of Si and O elements (Fig. 2C).

### 3.6. Antibacterial activity of TN-AgNPs

#### 3.6.1. MIC and MBC determination

**Fig. 3:**
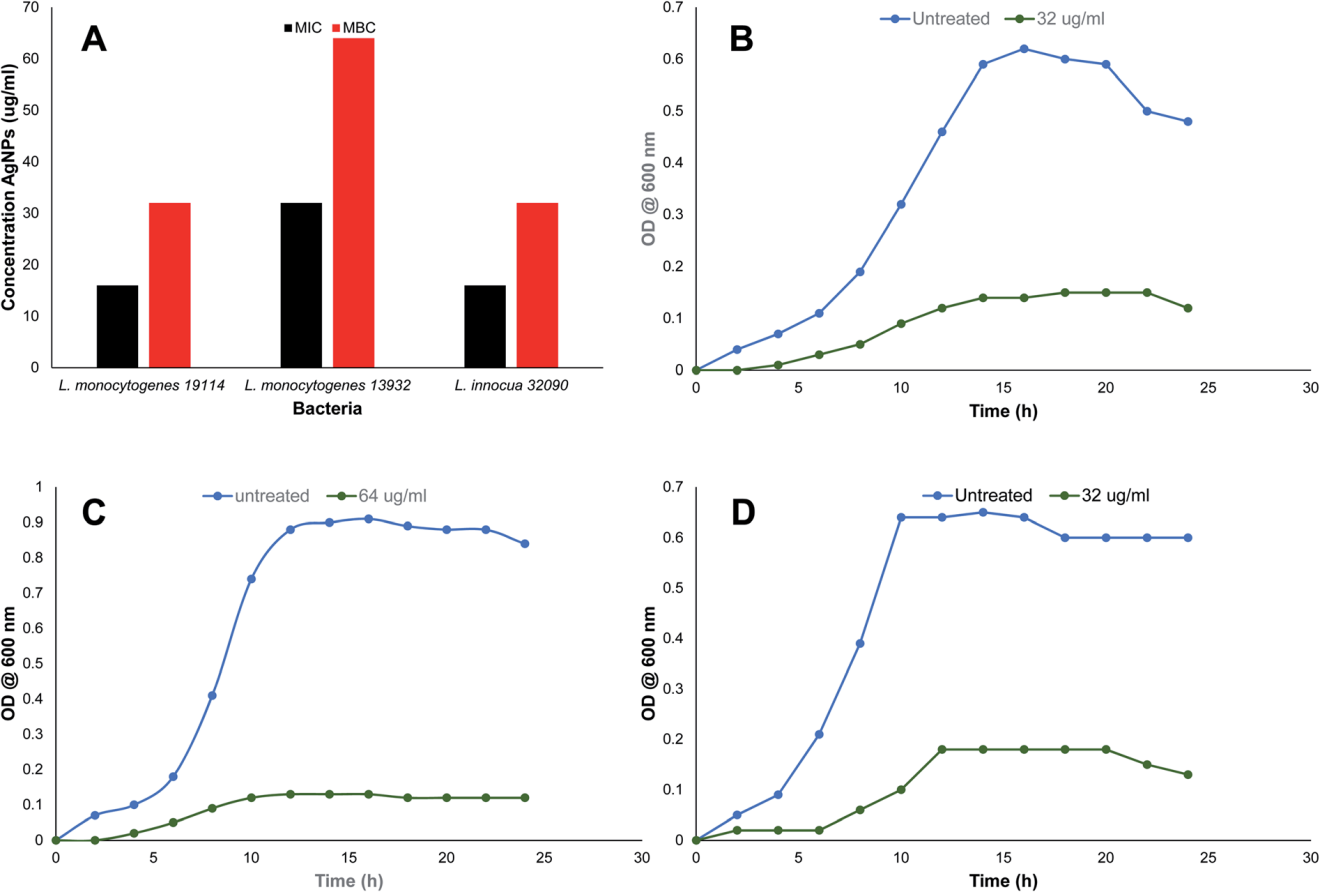
Antibacterial activity of TN-AgNPs against test strains of *Listeria* species. (A) MIC and MBC values in μg ml^−1^ of TN-AgNPs. Growth kinetics of (B) *Listeria monocytogenes* 11914, (C) *L. monocytogenes* 13932 and (D) *L. innocua* 33090.

TN-AgNPs were effective as antilisterial agents. The MBC is the lowest concentration that kills 100% of the bacterial load and does not show any viable growth on agar plates. The MBC for TN-AgNPs against *L. monocytogenes* ATCC 19114, *L. monocytogenes* ATCC 13932, and *L. innocua* 33090 was 32, 64, and 32 μg ml^−1^, respectively (Fig. 3A).

MIC values observed against all the three *Listeria* strains is presented in Fig. 3A. MIC values for TN-AgNPs against *L. monocytogenes* 19114 and *L. monocytogenes* 13932 was found to be 16 and 32 μg ml^−1^ while *L. innocua* demonstrated MIC of 16 μg ml^−1^. Our findings find support from the observations made with AgNPs synthesized from the outer peel extract of *Ananas comosus*. AgNPs demonstrated a slightly higher MIC and MBC values of 50 and 100 μg ml^−1^ respectively, against *L. monocytogenes*.^36^ Moreover, a similar observation was recorded earlier where AgNPs synthesized from the aqueous leaf extract of *Murraya koenigii* exhibited MICs as 32, 16, and 64 μg ml^−1^ against methicillin-resistant *S. aureus* (MRSA), *E. coli*, and ESβL-producing *E. coli*.^12^ In another study, AgNPs synthesized from the extract of *Phyla dulcis* were found effective against *E. coli* O157:H7, drug-resistant *S. typhimurium*, *L. monocytogenes* and *S. aureus*.^37^ The differences in the MIC and MBC values may be due to the intrinsic tolerance level of the strains used in the assay, variation in the shape and size of AgNPs and protocol used. Effect on bacterial was studied at 2×MICs while swimming and biofilm assays were performed using sub-MICs (0.5×MIC).

#### 3.6.2. Effect on growth kinetics of test *Listeria* strains

Twice of MIC (2×MIC) values were considered to study the effect of synthesized TN-AgNPs on the growth of *L. monocytogenes* 19114, *L. monocytogenes* 13932 and *L. innocua* 33090. Growth was examined every two hours for both AgNP treated and untreated (control) by measuring absorbance at 600 nm. Fig. 3B–D very clearly depicts the significant growth inhibitory activity of the TN-AgNPs against the all three test bacteria. Extent of growth inhibition was calculated in terms of percent reduction as compared to untreated control and 74.5, 86 and 70% growth inhibition was recorded against *L. monocytogenes* 19114, *L. monocytogenes* 13932 and *L. innocua* 33090, respectively, at their respective 2×MICs. Similarly, large-sized AgNPs bio-synthesized from extract of *Angelica keiskei* demonstrated effective bactericidal activity at 2×MIC against *L. monocytogenes*.^38^ The effect of AgNPs on the growth kinetics of drug-resistant bacteria have also been previously studied. A study found that there was 31.4%, 53.3%, and 85.6% reduction in growth of methicillin-resistant *S. aureus* (MRSA) in the presence of 8, 16, and 32 μg ml^−1^ AgNPs. Similarly, the treatment with same concentrations of AgNPs inhibited the growth of ESβL-producing *E. coli* by 21.3%, 57.4%, and 60.4%.^12^ Growth kinetics study was used to assess the antibacterial potential of *Captidis rhizome*-biosynthesized AgNPs against *E. coli* and *S. aureus*. Concentration-dependent decrease in the growth of the pathogens was recorded upon treatment with the biosynthesized AgNPs.^39^ Potent antibacterial property of AgNPs has been credited to the release of Ag^+^ ions, direct damage of cell membrane and/or generation of ROS.^17^

#### 3.6.3. SEM analysis of cell morphology

**Fig. 4:**
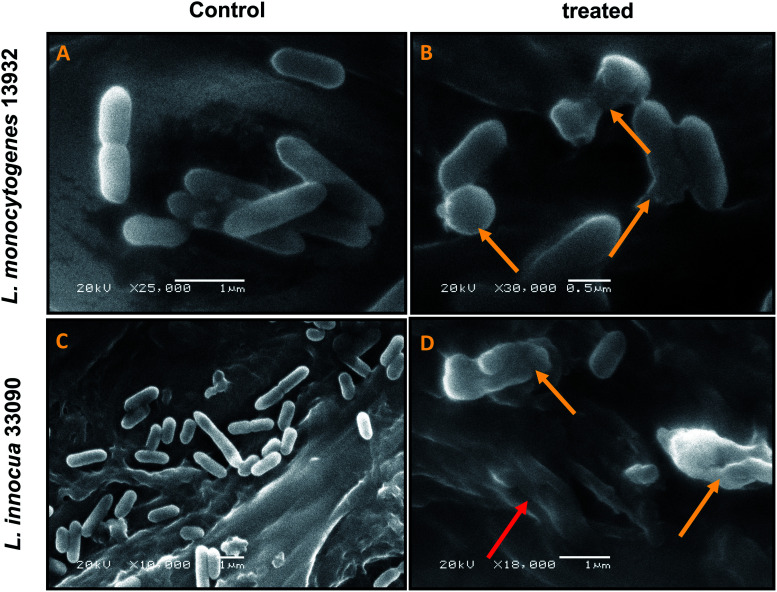
SEM analysis of cell morphology. (A) Untreated *L. monocytogenes* 13932 cells. (B) TN-AgNP treated (64 μg ml^−1^) cells of *L. monocytogenes*. (C) Untreated *L. innocua* 33090. (D) AgNP treated (32 μg ml^−1^) cells of *L. innocua* 33090. Yellow arrows indicate distortion of cells and red arrow shows clumping of cells.

SEM analysis of untreated cultures (control) and AgNPs treated cultures was undertaken to study the effect on cell morphology. The results presented in Fig. 4B and D show clumping of cells, distorted morphology due to rupturing of the cell membrane in cells treated with AgNPs. However, untreated (control) bacteria demonstrated regular cell morphology (Fig. 4A and C). SEM images clearly indicate at the incorporation of silver ions on the cell membrane leading to lysis of the target bacteria.

#### 3.6.4. Loss of cellular content

**Fig. 5:**
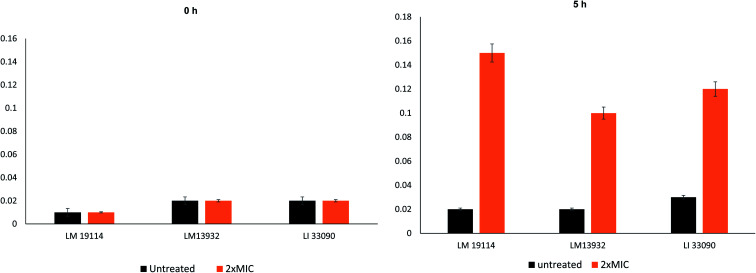
Cellular content release of test *Listeria* strains at time 0 h and 5 h.

Release of intracellular content from the bacteria is useful in determining the integrity of the cell membrane *i.e.* if the membrane of the bacteria is compromised release of the cytoplasmic constituents will be released out of the cell.^40^ Cell membrane has been suggested as one of the prime targets of the AgNPs, information on the loss of cellular content could be an important evidence in understanding the mechanism of action of these NPs. Release of cellular content in the treated and untreated cell filtrates was examined by reading absorbance at 260 nm. Treatment with 2×MICs of TN-AgNPs resulted in significant increase in the released cellular content (Fig. 5). The findings of the SEM analysis integrated with the observed release of cellular content, demonstrates that AgNPs interact strongly with the cells of the *Listeria* spp. leading to membrane damage, release of intracellular content resulting in cell death.

### 3.7. Swimming motility

**Fig. 6:**
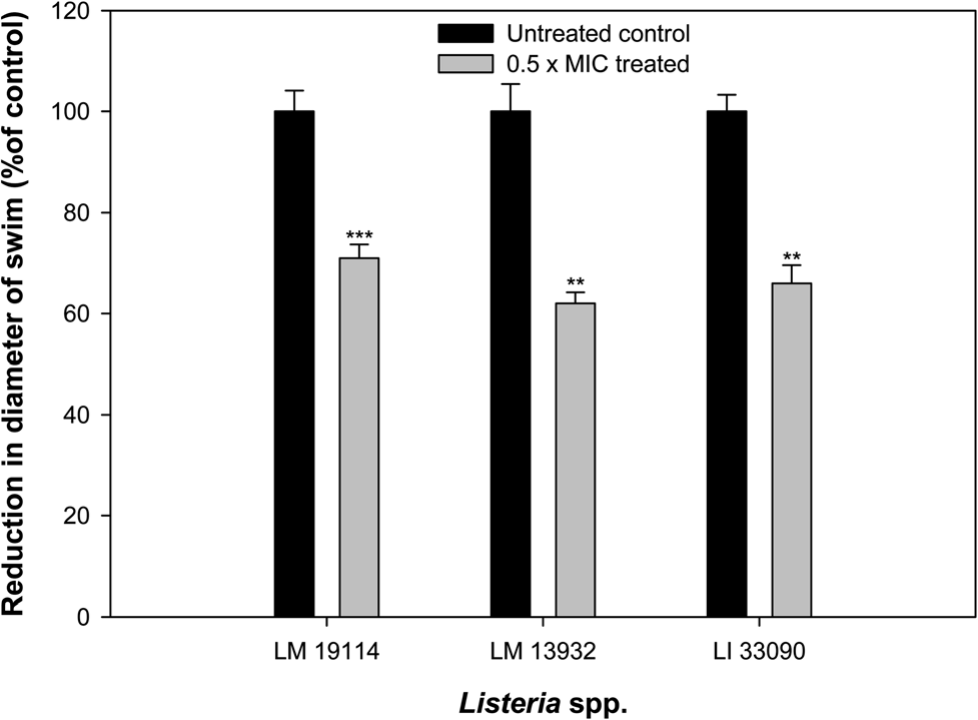
Effect of 0.5×MICs of TN-AgNPs on the swimming motility of test strains of *Listeria* species. Results are depicted as percentage reduction as compared to the untreated control. ** (*p* ≤ 0.05) and *** (*p* ≤ 0.01).

Influence of sub-MICs of synthesized TN-AgNPs was examined on the swimming behavior of test the *Listeria* strains. Swimming motility of all the three test pathogens was impaired significantly (*p* ≤ 0.05) at tested sub-MICs as compared to their untreated controls (Fig. 6). Swimming motility was reduced by 71, 62 and 66% in *L. monocytogenes* 19114 (8 μg ml^−1^), *L. monocytogenes* 13932 (16 μg ml^−1^) and *L. innocua* 33090 (8 μg ml^−1^), respectively. Reports have indicated about the positive role that motility plays in the formation of biofilm by *L. monocytogenes* as before attachment the bacteria has to travel to the surface of the host.^6^ Our observations showed significantly reduced motility in all the test strain as the AgNPs might be interfering with the flagellin synthesis and this could result in reduced biofilm formation in the under study bacteria.

### 3.8. Effect on biofilm formation

**Fig. 7:**
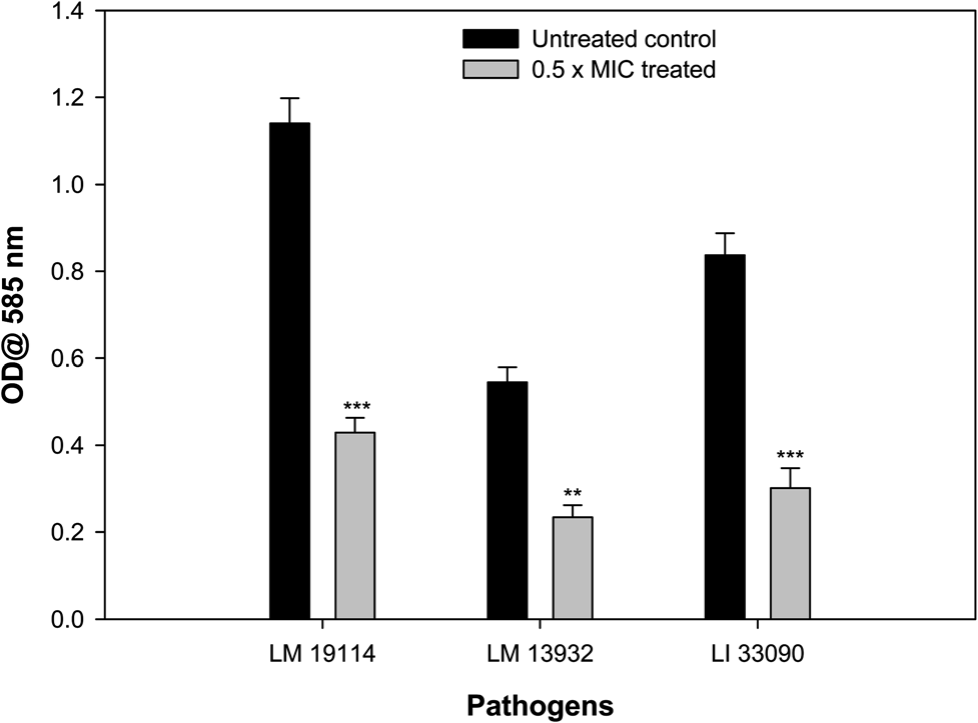
Effect of 0.5×MICs of TN-AgNPs on biofilm formation of test strains of *Listeria* species. Results are depicted as percentage reduction. ** (*p* ≤ 0.05) and *** (*p* ≤ 0.01).

Biofilm forming potential of the three test strains of *Listeria* species was assessed in the presence of their respective sub-MIC of TN-AgNPs. Biofilm formation in all the test strains was impaired significantly upon treatment with the TN-AgNPs as compared to their untreated controls (Fig. 7). There was 62% and 64% reduced biofilm formation in the presence of 8 μg ml^−1^ of TN-AgNPs in *L. monocytogenes* 19114 and *L. innocua* 33090, respectively. While, at 16 μg ml^−1^ concentration TN-AgNPs decreased the biofilm formation by 57% in *L. monocytogenes* 13932. The tested pathogens are proven biofilm formers that makes them dangerous as an infection causing bacteria leading to several health hazards and economic losses.^8^ Although there is no dearth of information on the antibacterial action of AgNPs but only few reports are available regarding biofilm inhibitory potential of AgNPs against *Listeria* species. In a recent report, AgNPs containing polyester surface exhibited effective inhibition of biofilm formed by *L. monocytogenes* at concentration of 500 ppm.^41^ The AgNPs have been wide reported for antibiofilm activity in literature. For instance, AgNPs synthesized using aqueous extract of *Withania somnifera* reduced the biofilms development of *S. aureus*, *S. mutans*, *P. aeruginosa*, and *S. typhimurium* by 40, 54, 48, and 50.72, respectively, compared their respective untreated controls.^27^ Moreover, nanoparticles of iron oxide, tin oxide, gold, copper and organosilane have also been shown to inhibit biofilm of *L. monocytogenes*.^42–46^ This is probably the first report on the inhibition of biofilm formation in *Listeria* species by green synthesized AgNPs.

### 3.9. Visualization of biofilm inhibition on glass coverslips

**Fig. 8:**
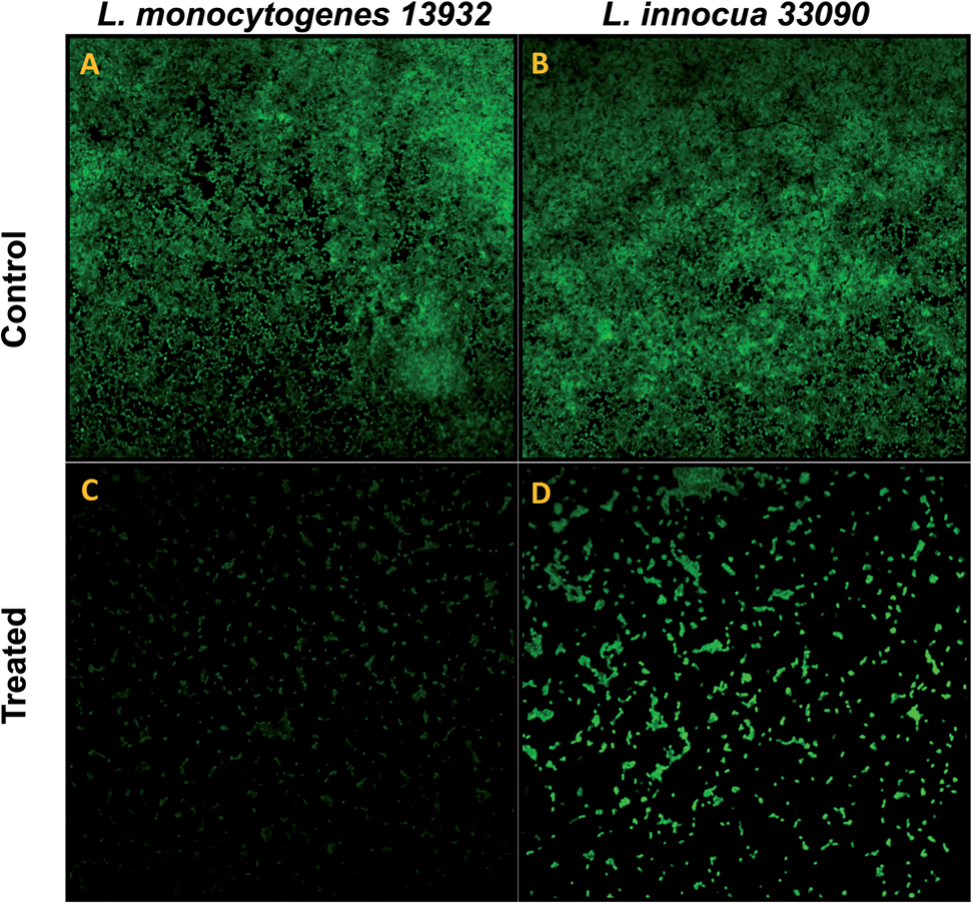
Confocal laser scanning microscopic (CLSM) analysis of biofilm inhibition (scale bar = 10 μm), stained with acridine orange. (A) Untreated (control) biofilm of *L. monocytogenes* 13932. (C) *L. monocytogenes* 13932 treated with 16 μg ml^−1^ of TN-AgNPs. (B) Untreated (control) biofilm of *L. innocua* 33090. (D) *L. innocua* 33090 treated with 8 μg ml^−1^ of TN-AgNPs.

**Fig. 9:**
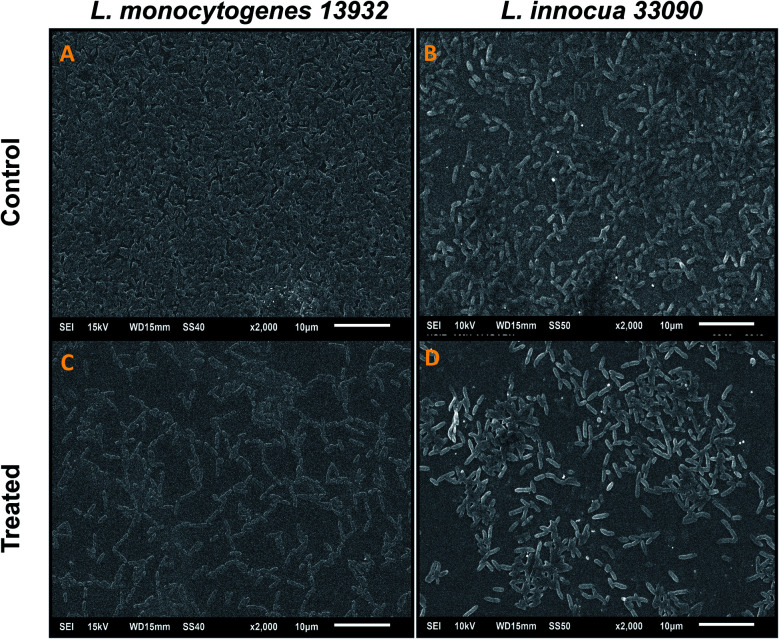
Scanning electron microscopic (SEM) analysis of biofilm inhibition. (A) Untreated (control) biofilm of *L. monocytogenes* 13932. (C) *L. monocytogenes* 13932 treated with 16 μg ml^−1^ of TN-AgNPs. (B) Untreated (control) biofilm of *L. innocua* 33090. (D) *L. innocua* 33090 treated with 8 μg ml^−1^ of TN-AgNPs.

Data obtained by the microtitre plate assay was further validated by examining biofilm formed on glass coverslips. SEM and CLSM images of biofilm of AgNPs treated (0.5×MIC) and untreated (control) were analyzed as depicted in Fig. 8 and 9. Scanning electron micrographs (Fig. 8A and B) show extensive formation of biofilm by untreated *L. monocytogenes* 13932 and *L. innocua* 33090. These untreated rod shaped cells are found embedded in dense EPS layer as clumped and clustered cell mass. On the contrary, micrograph of AgNP treated cells (Fig. 8c and d) clearly shows diminished colonization, less aggregation and reduced EPS. A disturbed biofilm architecture is observed in the AgNPs treated cells. Thus, SEM images provide a visual evidence about reduced biofilm formation in treated test strains of *Listeria* at sub-MICs. CLSM images also corroborate well with the findings of the quantitative data obtained for biofilm inhibition. Dense cell clusters in the untreated controls (Fig. 9a and b) were formed on the glass coverslips. While, in the presence of sub-MICs of AgNPs, cell clusters were greatly reduced and considerably less colonization of cells was observed (Fig. 9c and d).

### 3.10. Disruption of pre-formed biofilm

**Fig. 10:**
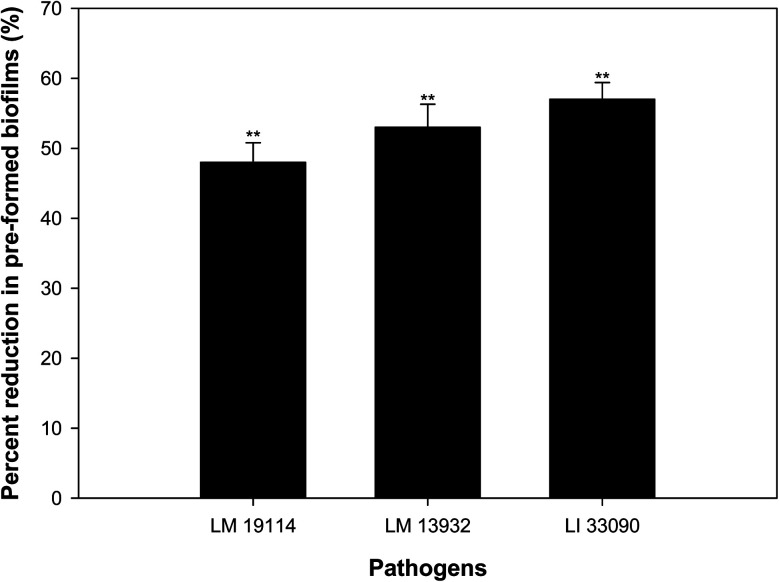
Inhibitory effect of sub-MICs (0.5×MIC) of TN-AgNPs on the pre-formed biofilms of test strains of *Listeria* spp. Results are depicted as percentage reduction in comparison to untreated control. ** (*p* ≤ 0.05).

Bacteria residing in biofilm mode are almost thousand times more tolerant to the action of antibiotics, disinfectants and detergents than the free living bacteria.^47^ Therefore, eradication of pre-formed biofilms is a difficult proposition. TN-AgNPs were assessed for their ability to disrupt pre-formed biofilms at sub-MICs (0.5×MIC). The results showed that at respective 0.5×MIC of TN-AgNPs, 48%, 53% and 58% reduction in the pre-formed biofilms of *L. monocytogenes* 19114, *L. monocytogenes* 13932 and *L. innocua* 33090 was recorded, respectively (Fig. 10). Biofilm cells are enclosed in matrix of EPS, which makes the cells resistant to the action of drugs, disinfectants, biocides and metal ions by preventing their entry.^48^ Significant drop in the pre-formed biofilms of all tested strains of *Listeria* upon treatment with TN-AgNPs revealed that the synthesized NPs could successfully breach the EPS barrier and disrupt established biofilms. This probably the first report on the disruption of pre-formed biofilms of *Listeria* species by AgNPs synthesized from plant material.

### 3.11. Effect on ROS generation

**Fig. 11:**
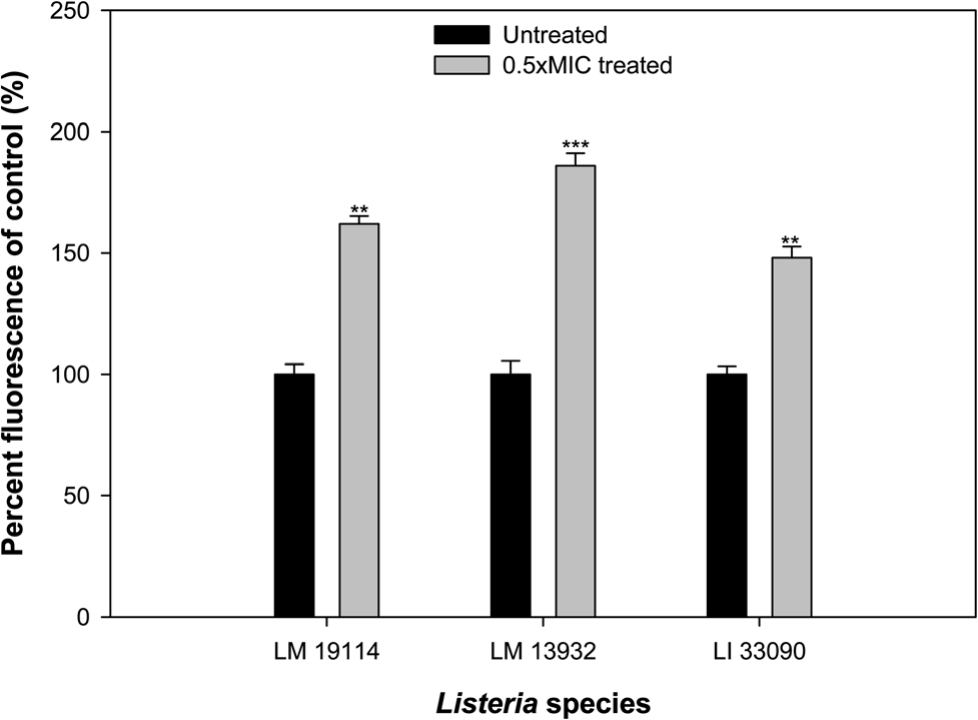
TN-AgNPs induced ROS generation in the sub-MICs treated and untreated test bacteria. ** (*p* ≤ 0.05) and *** (*p* ≤ 0.01).

The potential of sub-MICs of TN-AgNPs to induce ROS production in the three strains of *Listeria* was assessed using fluorescent probe DCFH-DA. Significant amount of ROS generation was recorded in AgNPs treated cells as compared to their untreated controls (Fig. 11). ROS levels increased by 62% in *L. monocytogenes* 19114, while an upsurge of 86% was recorded in other test strain of *L. monocytogenes* (LM 13932). *L. innocua* 33090 exposed to sub-MIC (8 μg ml^−1^) of TN-AgNPs exhibited 48% elevated ROS levels. Production of ROS in nanoparticle treated cells surpasses the capacity of antioxidant defense system leading to oxidative stress that starts lipid peroxidation. As a result, cell death occurs due to the damage to cell membrane.^49^ Thus, ROS generation by AgNPs is a key mechanism in the inhibition of biofilm.

### 3.12. Wound healing assay: cell migration analysis

**Fig. 12:**
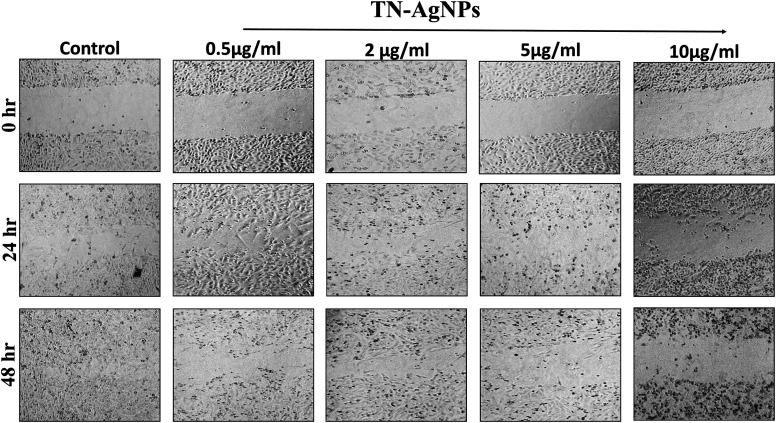
Wound healing assay to assess the migration of TN-AgNPs treated and untreated MCF-7 cells through scratch assay. Images were captured at 0, 24 and 48 h.

Wound healing or scratch assay is a robust and most commonly used method to probe cell migration.^50^ In this study, MCF-7 cells were subjected to wound healing assay under the influence of different concentration of AgNPs (0.5–10 μg ml^−1^). Scratch plate assay showed that the AgNPs at all tested concentration impaired the cell migration effectively (Fig. 12). Further, our result showed concentration and time dependent inhibition on the migration of cells. At lower concentration (0.5–2 μg ml^−1^) of AgNPs, less cytotoxicity and inhibition of migration was observed. However, its higher concentration (5–10 μg ml^−1^) causes more cytotoxicity and distinct inhibition of migration of cells. As cytoskeleton rearrangements are crucial for cell migration, it is noteworthy to speculate that AgNPs might interfere with this process reaffirming earlier report.^51^ Since cell division requires cytoskeleton rearrangement, effect of AgNPs on cell proliferation can also be seen in Fig. 12. At 5 and 10 μg ml^−1^ AgNPs after 48 hours, reduction in cell number was observed as compared to untreated control. Tested concentrations of the synthesized AgNPs significantly effect cell proliferation and migration. Our results corroborate well with the previous literature reporting the inhibitory effect of nanoparticles on the migration of cancer cells.^52^ Moreover, our cytotoxicity analysis revealed the effectiveness of AgNPs between 0.5–10 μg ml^−1^, which is in line with previous studies.^53,54^ From the present findings, it is envisaged that not only the TN-AgNPs will speed up the healing process but also prevents the colonization of bacteria in wounds as it possesses both antibacterial and antibiofilm properties.

## 4. Conclusion

The study showcases the efficiency of extract of *in vitro* grown *T. nilotica* shoot to fabricate silver nanoparticles (AgNPs) without using any hazardous chemical. The characterized AgNPs demonstrated significant inhibition of bacterial growth of three test strains of *Listeria*. SEM images demonstrated that the AgNPs caused distortion of cell morphology leading to cell death. Further, the synthesized AgNPs significantly impaired biofilm formation as well as disrupted pre-formed biofilms at sub-MICs. Finding of the biofilm inhibition were also confirmed by SEM and CLSM analysis. Interaction of bacterial cells in and sub-MICs of AgNPs leads to production of excessive ROS causing cell death and subsequently inhibition of biofilms. In addition to the antibacterial and antibiofilm action, synthesized AgNPs exhibited effective wound healing potential. Therefore, the findings of investigation advocates that the synthesized AgNPs can be exploited as an alternative or supplementary strategy to mitigate the contamination and infection caused by *L. monocytogenes*.

## Conflicts of interest

The authors declare no conflict of interest.

## Supplementary Material
